# Characterizing the Indoor-Outdoor Relationship of Fine Particulate Matter in Non-Heating Season for Urban Residences in Beijing

**DOI:** 10.1371/journal.pone.0138559

**Published:** 2015-09-23

**Authors:** Lihui Huang, Zhongnan Pu, Mu Li, Jan Sundell

**Affiliations:** 1 Key Laboratory of Subsurface Hydrology and Ecological Effects in Arid Region, Ministry of Education, School of Environmental Science and Engineering, Chang’an University, Xi’an, 710054, China; 2 Institute of Built Environment, Department of Building Science, Tsinghua University, Beijing, 100084, China; Peking University, CHINA

## Abstract

**Objective:**

Ambient fine particulate matter (PM_2.5_) pollution is currently a major public health concern in Chinese urban areas. However, PM_2.5_ exposure primarily occurs indoors. Given such, we conducted this study to characterize the indoor-outdoor relationship of PM_2.5_ mass concentrations for urban residences in Beijing.

**Methods:**

In this study, 24-h real-time indoor and ambient PM_2.5_ mass concentrations were concurrently collected for 41 urban residences in the non-heating season. The diurnal variation of pollutant concentrations was characterized. Pearson correlation analysis was used to examine the correlation between indoor and ambient PM_2.5_ mass concentrations. Regression analysis with ordinary least square was employed to characterize the influences of a variety of factors on PM_2.5_ mass concentration.

**Results:**

Hourly ambient PM_2.5_ mass concentrations were 3–280 μg/m^3^ with a median of 58 μg/m^3^, and hourly indoor counterpart were 4–193 μg/m^3^ with a median of 34 μg/m^3^. The median indoor/ambient ratio of PM_2.5_ mass concentration was 0.62. The diurnal variation of residential indoor and ambient PM_2.5_ mass concentrations tracked with each other well. Strong correlation was found between indoor and ambient PM_2.5_ mass concentrations on the community basis (coefficients: r≥0.90, p<0.0001), and the ambient data explained ≥84% variance of the indoor data. Regression analysis suggested that the variables, such as traffic conditions, indoor smoking activities, indoor cleaning activities, indoor plants and number of occupants, had significant influences on the indoor PM_2.5_ mass concentrations.

**Conclusions:**

PM_2.5_ of ambient origin made dominant contribution to residential indoor PM_2.5_ exposure in the non-heating season under the high ambient fine particle pollution condition. Nonetheless, the large inter-residence variability of infiltration factor of ambient PM_2.5_ raised the concern of exposure misclassification when using ambient PM_2.5_ mass concentrations as exposure surrogates. PM_2.5_ of indoor origin still had minor influence on indoor PM_2.5_ mass concentrations, particularly at 11:00–13:00 and 22:00–0:00. The predictive models suggested that particles from traffic emission, secondary aerosols, particles from indoor smoking, resuspended particles due to indoor cleaning and particles related to indoor plants contributed to indoor PM_2.5_ mass concentrations in this study. Real-time ventilation measurements and improvement of questionnaire design to involve more variables subject to built environment were recommended to enhance the performance of the predictive models.

## Introduction

The rapid urbanization and industrialization result in the recent nationwide ambient fine particle pollution (haze episodes) in Chinese urban areas. Fine particulate matter (PM_2.5_) is a complicated mixture of solid and liquid particles that vary in number, size, shape, surface area, chemical composition, solubility and origin [1, 2]. Exposure to toxic components in ambient PM_2.5_ is associated with a wide spectrum of adverse respiratory and cardiovascular health effects[3–12], and even with low birth weight and increased risk of preterm birth [13, 14]. Therefore, fine particle pollution in ambient air is currently a major public health concern in China, and is driving increasing research interest [12, 15–17].

Chinese urban residents spend nearly 90% of lifetime indoors, and of that, 70% is in residential indoor microenvironments[18]. As a result, exposure to airborne PM_2.5_ primarily occurs indoors. Ambient PM_2.5_ penetrate indoors through the ventilation system, the building envelope and cracks [19–21]. The outdoor-indoor transit of ambient particles can alter PM chemical and physical properties such as mass concentrations, size-distribution and composition [20, 22–24]. For example, the fraction of ambient PM_2.5_ that can penetrate indoors and remain suspended (defined as infiltration factor, *F*_inf_) largely vary from 35% to 95% in the literature[19, 25–32]. Particles in the accumulation mode have higher *F*_inf_ values than particles of other size bins [24, 26]. On the other hand, particles generated by indoor sources may also significantly contribute to indoor PM_2.5_. The primary indoor PM_2.5_ sources include environmental tobacco smoke (ETS), home cleaning and heat/combustion activities[21, 33]; whereas the secondary sources include, but not limited to, ozonolysis of unsaturated hydrocarbons and oxidation of aromatics [30, 34, 35].

Due to the properties alteration during infiltration process and the presence of indoor sources, the diurnal variation pattern of indoor and outdoor PM_2.5_ concentrations may significantly differ. The ratio of indoor to outdoor PM_2.5_ mass concentrations (defined as I/A in this article) can be greater than unity sometimes. It is thus uncertain if central-site PM_2.5_ mass concentrations are good surrogates of exposure to ambient PM_2.5_. Given such, it is necessary to characterize the relationship between indoor and outdoor PM_2.5_ concentrations as part of fine particle exposure assessment, which has not been well studied in China.

The objective of this study is to characterize the indoor-ambient relationship of PM_2.5_ mass concentrations in non-heating season for urban residences in Beijing. We concurrently collected real-time indoor and outdoor PM_2.5_ mass concentrations for 41 urban residences in Beijing in the non-heating season. Infiltration factor of ambient PM_2.5_ and its determinants have been discussed in a companion paper. In this article, we compared the diurnal variation of indoor and ambient PM_2.5_ mass concentrations as well as I/A. Further, we explored the predictors of indoor PM_2.5_, which suggested the likely indoor PM_2.5_ sources in the tested urban residences during the monitoring period. The results and the mechanisms driving the observations are discussed in this article.

## Materials and Methods

Fudan University’s ethical review board approved the study protocol (IRB00002408 & FWA00002399). The field tests were conducted based upon permissions from the property owners. A written consent was obtained from the participants prior to the field visit. We also implemented sampling at several outdoor sites in this study, in order to compare the measurements of our light-scattering devices to those of Chinese National Environmental Quality Monitoring (EQM). Permissions were obtained from Citic Group (the owner of the site close to Agriculture Exhibition Center EQM station), Department of Building Science, Tsinghua University (the owner of the site close to Wanliu EQM station), and the private land owners of other sites.

### Buildings sampled

The families that participated in this study were from a large cohort, which was recruited for a public health research project. The randomly selected cohort consisted of families in Beijing having 1–8 years old children. We contacted the parents in each family via telephone, informed them the research objective, and recruited the families that were willing to join in. Forty-one families participated in this study. These residences are located in 6 districts in Beijing: 17 in *Haidian*, 5 in *Xicheng*, 3 in *Fengtai*, 1 in *Shijingshan*, 1 in *Changping* and 14 in *Chaoyang*. Questionnaires were administrated to the occupants during each visit to gather demographic and social-economic information, building characteristics, lifestyle information and time-activity pattern.

The dwellings tested in this study are relatively young (~50% less than 10-yr old). The occupants were also the property owners in 89% of the tested dwellings. All residences are multistory (≤7 stories, 39% of residences) or high-rise type (> 7 stories, 61% of residences). Mechanical ventilation is employed by only 1 residence, and others ventilate the indoor spaces through opening windows. Radiators are used for heating in 91% of the dwellings, whereas floor heating is used in the others. Central air conditioning is used for cooling in 2 dwellings, whereas split type air conditioning is used for cooling in the others. According to the time-activity records, ~60% families turned on air conditioner (AC) for 1–3 hours during the monitoring periods, and the windows were open when AC was not in operation.

**Fig 1:**
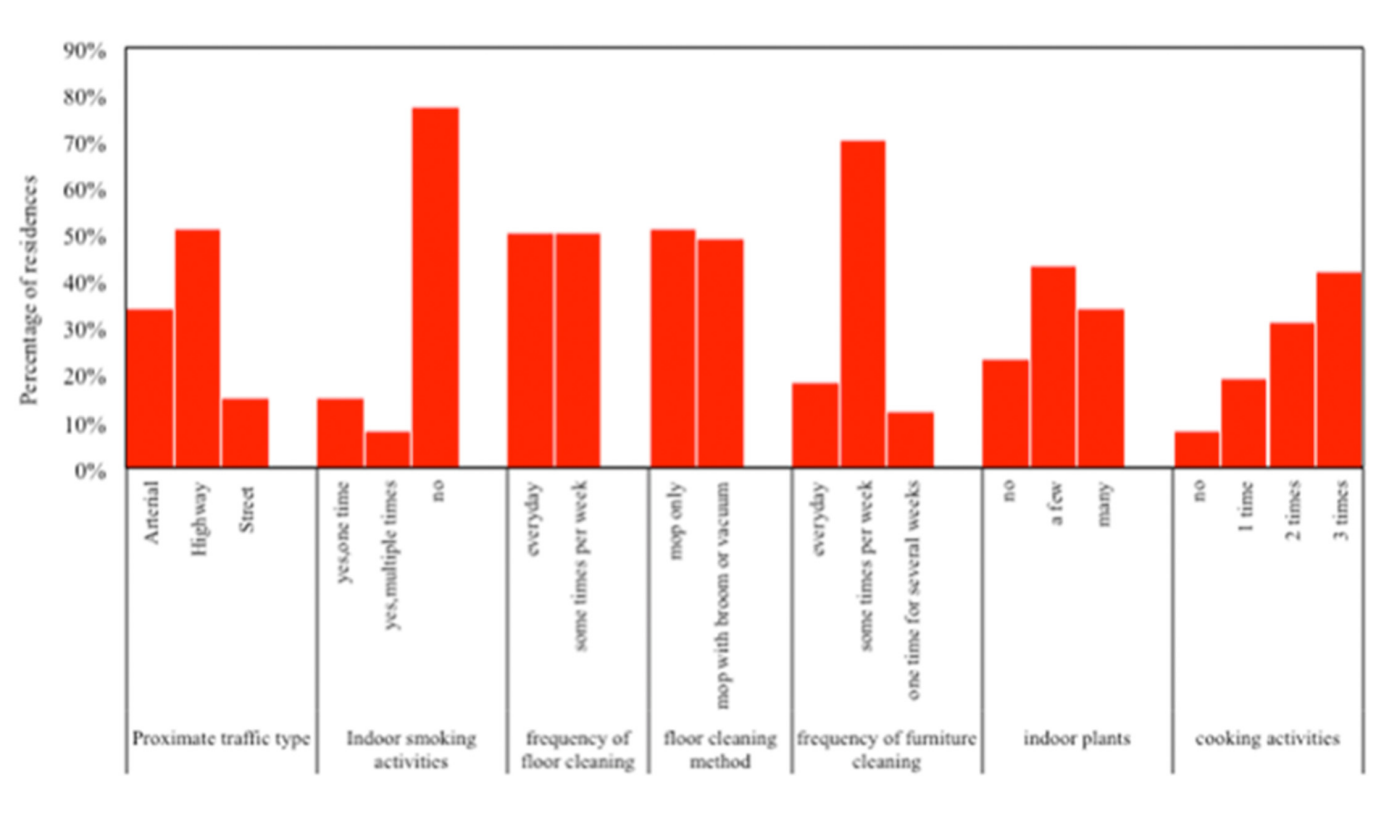
The information of variables subject to building characteristics and human behavior.

Variables subject to building characteristics and human behavior that are associated with indoor PM_2.5_ sources were of particular interests in the subsequent analysis. These variables included t*he type of traffic route around residence*, *distance to the road*, *indoor smoking activities during the monitoring period*, *cooking activities during the monitoring period*, f*requency of floor cleaning*, *floor cleaning method*, *frequency of furniture cleaning*, *indoor plants* and *number of occupants*. Traffic emission is one of the important sources of indoor PM_2.5_ [23, 36]. Therefore, variables subject to traffic conditions may be important predictors of indoor PM_2.5_. Three types of traffic routes can be found proximate to the tested residences: expressways or arterial roads (ring roads of Beijing), local highways and quiet streets (Fig 1). The distances between each residence and the proximate road were measured and grouped into 4 quartiles (1st quartile: ≤ 193 m, 2nd quartile: 194–270 m, 3rd quartile: 271–425 m, 4th quartile: >425 m). Indoor smoking activities occurred in 23% of the tested dwellings during the monitoring periods (Fig 1). Cleaning activities can lead to resuspension of particles, which are also important sources of indoor PM_2.5_ [37, 38]. Thus, we collected the information of indoor cleaning, which included *frequency of floor cleaning*, *method of floor cleaning* and *frequency of furniture cleaning* (as shown in Fig 1). Cooking is another important source of indoor PM_2.5_, particularly ultrafine particles [39]. The cooking times during the monitoring periods were presented in Fig 1. Indoor plants can be another predictor of indoor PM_2.5_, given resuspension of soil particles and submicron particles generation via biogenic VOCs-ozone reactions [35]. Fig 1 presents the information of indoor plants in the tested dwellings. In addition, we considered the variable, *number of occupants*, given that human activities indoors are associated with particle generation. The maximum occupant number was 7, whereas the minimum was 0. The dwellings with 2 to 6 occupants accounted for 6%, 33%, 14%, 28% and 11% of total residences, respectively.

### Field measurements

The field visits were implemented from July 1st to September 8^th^, 2013. A light-scattering SidePAK^TM^ personal aerosol monitor (AM510, TSI Inc.) was deployed at a height of 1.5 m (breathing zone) in the living room of each residence to collect the hourly real-time indoor PM_2.5_ mass concentrations for consecutive 24 hours. The 24-h sampling period is frequently employed in exposure measurement and assessment, given that the data can reflect the daily average exposure levels of pollutant concentrations[40, 41]. As part of the quality assurance and quality control (QAQC) procedures, the photometers were calibrated every month against gravimetric measurements. Additionally, the standard deviation (SD) of the readings obtained by the collocated three photometers was 3.9±1.4%, which was evaluated in the laboratory prior to the field visits.

Due to constraints on the placement of monitors outdoors and availability of power, we did not collect concurrent real-time residential outdoor PM_2.5_ mass concentrations using the above devices, but obtained the real-time ambient PM_2.5_ mass concentrations during the monitoring periods from the Chinese National Environmental Quality Monitoring (EQM) Stations that were closest to each residences. These stations served for central-site fixed monitoring in our study. TEOM 1405F (Thermo Fisher Scientific Inc., MA, US) are employed at each EQM station to measure ambient PM_2.5_ mass concentrations. These instruments are routinely calibrated against gravimetric measurements. The distances between each residence and the closest monitoring stations are presented in Table A in the S1 File. We were concerned of the potential errors when using central-site outdoor PM_2.5_ mass concentrations as the surrogate of residential outdoor counterpart, despite the fact that extensive studies have suggested ambient fixed site monitoring to be reasonable surrogates of residential outdoor monitoring with respective to PM_2.5_ [42, 43]. To assess this uncertainty, we measured PM_2.5_ mass concentrations using the photometers at outdoor sites where the devices can be securely placed and there was power supply. The test sites had variable distances to the closest EQM stations. We compared the data obtained by the photometers to those provided by the closest EQM stations. As shown in Table B in S1 File, strong Pearson correlation was found (coefficient: 0.86–0.95), and the differences between the two measurements did not significantly differ from zero when the distances were within either 5.5 or 8 km (p>0.50, paired sample *t*-test). Note: the distances between 76% of the residences and the nearest EPA stations are within 5.5 km, while the distances for the remaining residences are within 7.0 km (Table A in S1 File). The results indicated that it was reasonable to use ambient fixed site monitoring as the surrogate of residential outdoor monitoring with respective to PM_2.5_ in our study.

We also collected the ambient criteria air pollutants (i.e. O_3_, NO_2_ and SO_2_) concentration data and meteorological data in this study. The hourly real-time concentrations of O_3_, NO_2_ and SO_2_ in ambient air were provided by the EQM stations that are closest to each residence. Daily average outdoor meteorological data were provided by *China Meteorological Data Sharing Service System*. Real-time indoor temperature and RH values were recorded by Telaire 7001 monitor (Telaire Inc., USA), which was connected to a HOBO U12 data logger (Onset, USA) for data logging.

### Data analysis

Descriptive statistical analyses were conducted for ambient and indoor PM_2.5_ mass concentrations, I/A, ambient criteria air pollutants concentrations and meteorological data. A sampling day (24-hour) was divided into 4 sessions: morning (06:00–12:00), afternoon (12:00–18:00), evening (18:00–0:00) and night (0:00–6:00). The diurnal variation of indoor and ambient PM_2.5_ mass concentrations, I/A and criteria air pollutant concentrations were characterized. Due to the large day-to-day variability of pollutant concentrations, the original real-time data obtained in each residence were normalized prior to the diurnal variation characterization. The normalization procedure was as follows: the 24-h average values were set as the reference values with respect to each residence, and the hourly values were obtained by dividing the original hourly data by the 24-h average values.

The associations between indoor and ambient PM_2.5_ mass concentrations were examined using Pearson correlation analysis and regression analysis with ordinary least square (OLS) method, respectively. There were residences in which smoking activities occurred during the monitoring periods (smoking families). Given such, the data of all families were involved in the analyses first, and then the analyses were conducted again after excluding the data from the smoking families.

Predictive statistical models were built to characterize the variables that were associated with indoor and ambient PM_2.5_ mass concentrations. Regression analysis with OLS method was employed. The variables considered in the predictive model for ambient PM_2.5_ mass concentrations included the concentrations of ambient O_3_, NO_2_ and SO_2_. The variables considered in the predictive model for indoor PM_2.5_ mass concentrations included *proximate traffic type*, *distance to road*, *indoor smoking activities*, *cooking activities*, *indoor cleaning activities*, *indoor plants* and *criteria air pollutant concentrations*. The predictive models of indoor PM_2.5_ were built for all families and the non-smoking families, respectively. It is necessary to note that neither PM_2.5_ mass concentration data nor criteria air pollutant concentration data complied with normal or log-normal distribution (shown in Results section). However, it is still reasonable to apply the regression models to our data (N > 30) according to central limit theorem. The statistical analyses in this study were conducted with Statistical Analysis System (SAS v9.3, SAS Inc., Cary, NC). The significance level was 0.05 unless otherwise specified.

## Results

### Concentrations of pollutants

#### Ambient and indoor PM2.5

The field sampling was implemented in the non-heating season. The mean ambient and indoor temperatures over the 24-h sampling period were in the range of 22.2–30.7°C and 25.6–33.2°C, respectively. The maximum and minimum hourly indoor temperatures were 47.2°C and 21.2°C, respectively. The hourly indoor RH was 19.1–79.9%, whereas the mean values over the 24-h sampling periods were 41.6–77.2% and 44–91% for indoor and ambient RH, respectively.

Descriptive statistics of ambient and indoor PM_2.5_ mass concentrations are summarized in Table 1. The maximum hourly ambient PM_2.5_ mass concentration in this study was 280 μg/m^3^, and the minimum was 3 μg/m^3^ (Table 1). The ambient concentrations over the 24-h sampling period were in the range of 15–165 μg/m^3^ with mean and median of 69 and 62 μg/m^3^, respectively (Table 1). The ambient PM_2.5_ concentration data did not comply with normal or log-normal distribution (p<0.001, Shapiro-Walk, shown in Figure A in S1 File). The means and medians of hourly and 24-h average ambient PM_2.5_ mass concentrations met the health guideline suggested by *Chinese National Ambient Air Quality Standard* (GB 3095–2012), 75 μg/m^3^. However, 38% of the hourly observations were still above the guideline.

**Table 1:** Descriptive statistics of the concentrations of pollutants.

Pollutant	Unit	data category	N^a^	Mean	SD	Min	25%	Median	75%	Max
**Indoor PM**_**2.5**_	μg/m^3^	hourly	792	44	35	4	15	34	63	193
		daily average	33	44	28	9	22	36	65	119
**Ambient PM**_**2.5**_	μg/m^3^	hourly	792	69	49	3	30	58	96	280
		daily average	33	69	38	15	36	62	91	165
**I/A (PM**_**2.5**_ **)**		hourly	792	0.63	0.27	0.12	0.48	0.6	0.74	3.3
		daily average	33	0.63	0.15	0.31	0.54	0.62	0.73	0.98
**O**_**3**_	μg/m^3^	hourly	825	78	67	1	14	67	121	285
		daily average	35	76	30	18	54	77	104	123
**NO**_**2**_	μg/m^3^	hourly	816	51	25	8	31	47	68	143
		daily average	34	52	13	17	41	52	61	76
**SO**_**2**_	μg/m^3^	hourly	825	7	6	1	3	6	9	60
		daily average	35	8	4	3	5	7	9	19

a. Valid ambient and indoor PM_2.5_ mass concentrations were simultaneously available for 33 residences.

The maximum hourly indoor PM_2.5_ mass concentration in this study was 193 μg/m^3^, and the minimum was 4 μg/m^3^ (Table 1). The indoor concentrations over the 24-h sampling period were in the range of 9–119 μg/m^3^ with mean and median of 44 and 36 μg/m^3^, respectively (Table 1). Same as the ambient PM_2.5_ data, the indoor PM_2.5_ concentration data were neither normal nor log-normal distributed (p<0.001, Shapiro-Walk, shown in Figure B in S1 File).

**Fig 2:**
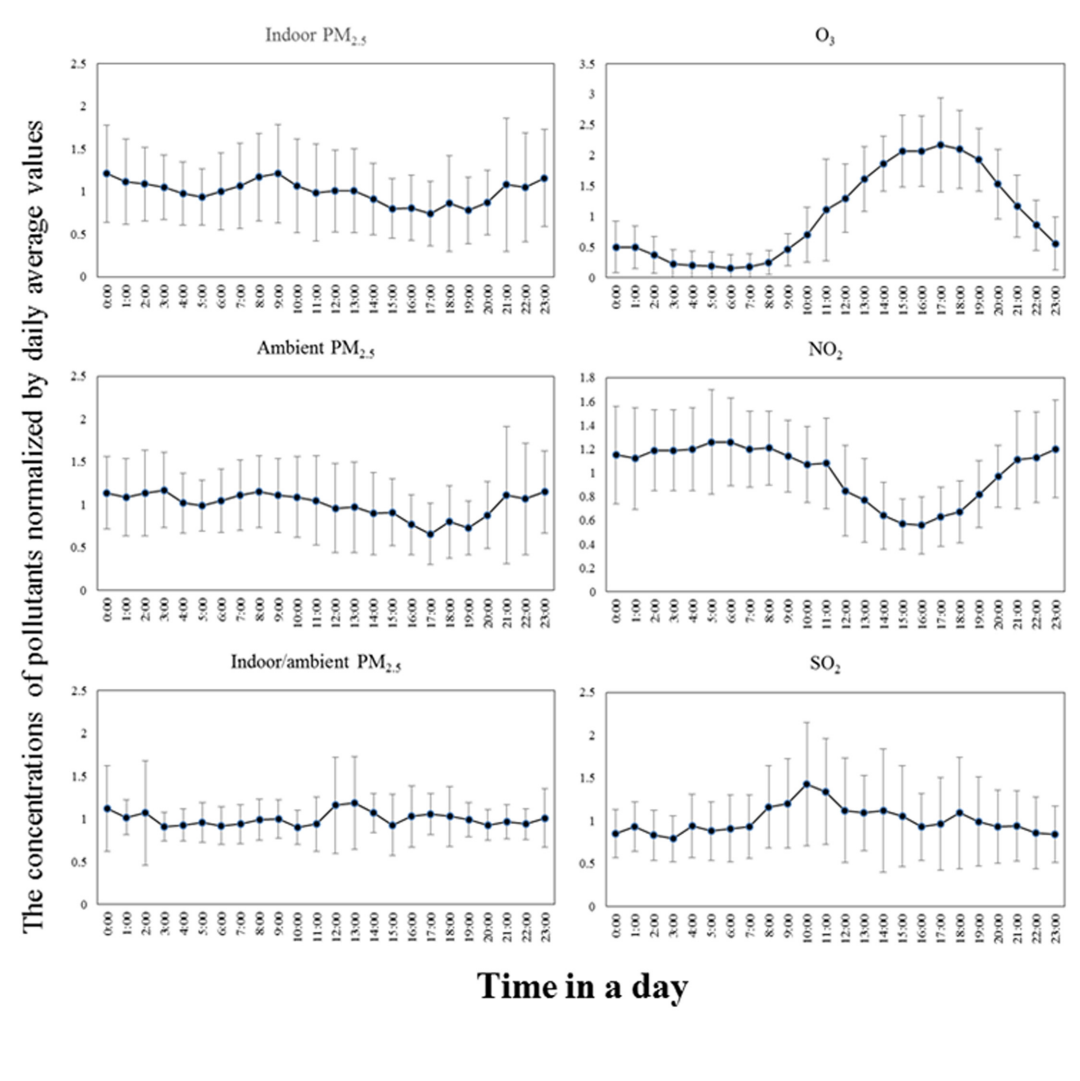
Diurnal variation of pollutant concentrations. (Note: error bars in the Fig stand for standard deviation of the normalized pollutant concentrations).

The diurnal variation of ambient PM_2.5_ concentrations was shown in Fig 2. It can be discerned that: a) the concentrations started dropping after 8:00, and fell below the average values over the 24-h sampling period in the afternoon; b) there was noticeable concentration increase in the evening (19:00–23:00), and the concentrations reached the highest levels at night and in early morning (21:00–10:00); c) a concentration decrease was found between 3:00 and 5:00, and then a slight concentration increase can be discerned in 5:00–8:00. It is also found that the diurnal variation of residential indoor PM_2.5_ concentrations tracked the ambient counterpart well (Fig 2).

#### Criteria air pollutants

In this study, the hourly ambient O_3_ concentrations were in the range of 1–285 μg/m^3^ with mean and median of 78 and 67 μg/m^3^, respectively (Table 1). The relatively high O_3_ episodes were expected, given the intense photochemical reactions in atmosphere in the summer. Of the hourly observations, 5% exceeded the health guideline suggested by GB 3095–2012 (200μg/m^3^). The hourly ambient NO_2_ concentrations were in the range of 8–143 μg/m^3^ with mean and median of 51 and 47 μg/m^3^, respectively (Table 1). No observations were above the health guideline of GB 3095–2012 (200μg/m^3^). Fig 2 shows the diurnal variation of ambient NO_2_ and O_3_ concentrations. Ambient O_3_ concentrations increased since 8:00 in the morning and reached the highest values at 15:00–18:00 in the afternoon. Contrary to O_3_, ambient NO_2_ concentrations started falling since 8:00 and reached the lowest values at 14:00–17:00. The reverse trends of ambient O_3_ and NO_2_ concentrations are expected, given that NO_2_ is the precursor of ground-level O_3_ in atmosphere and thus increasing O_3_ is accompanied by decreasing NO_2_ [44]. The hourly concentrations of ambient SO_2_ were 1–60 μg/m^3^ with mean and median of 7 and 6 μg/m^3^ (Table 1). Coal combustion for heating is the major source of ambient SO_2_ in Beijing. The sampling was conducted in the non-heating season, and thus the low ambient SO_2_ concentrations were within expectation. The criteria air pollutant concentration data did not comply with normal or log-normal distribution (p<0.001, Shapiro-Walk, shown in Figures C-E in S1 File).

### Relationship between indoor and ambient PM_2.5_ mass concentrations

As shown in Table 2, there was significant and strong positive correlation (r≥0.90, p<0.0001) between indoor and ambient PM_2.5_ mass concentrations over the 24-h sampling period as well as the four sessions. The results indicated that the indoor and ambient PM_2.5_ mass concentrations in this study highly correlated with each other on the community basis. Excluding the smoking families was not found to weaken the strong correlation (Table 2). Nonetheless, the correlation coefficients varied by single residences. No significant correlation was observed for one residence, moderate correlation (0.5≤r<0.8) for 7 homes, and strong correlation (r≥0.8) for the other homes. Of the 8 residences, the occupants from only 2 dwellings reported smoking activities indoors during the monitoring periods. Smoking was thereby not the major cause for the weak correlation. The leading reasons, however, cannot be identified through the questionnaire in this study.

**Table 2:** Pearson correlation between indoor and ambient PM_2.5_ mass concentrations.

	24-h period	Morning (6:00–12:00)	Afternoon (12:00–18:00)	Evening (18:00–0:00)	Night (0:00–6:00)
**Including all families**					
**Coefficient**	0.91	0.92	0.90	0.92	0.92
***p*-value**	<0.0001				
**Excluding smoking families**					
**Coefficient**	0.92	0.92	0.90	0.94	0.93
***p*-value**	<0.0001				

The linear regression analysis results are shown in Table 3. Ambient PM_2.5_ mass concentrations explained >80% variance of the indoor counterparts (Table 3). In addition, the variance explained by the ambient PM_2.5_ data over the 24-h sampling period (91%) was higher than that explained by the hourly real-time data (84%). The difference was reasonable, because the hourly indoor data were more sensitive to indoor PM_2.5_ concentration spikes due to emissions from indoor sources than the average data over the 24-h sampling period. Similar to the Pearson correlation analysis, excluding the smoking families did not significantly change the linear regression analysis results as well (Table 3). As stated earlier, there were 8 residences in our study, in which indoor and ambient PM_2.5_ mass concentrations did not strongly correlate with each other. The variance of indoor PM_2.5_ mass concentrations explained by ambient counterpart was ≤ 60% for the 8 residences, while it was ≥65% for the remaining residences (data not shown here).

**Table 3:** Regression analysis of indoor and ambient PM_2.5_ mass concentrations.

	Indoor PM_2.5_ (including all families)		Indoor PM_2.5_ (excluding smoking families)	
**Ambient PM**_**2.5**_ **(hourly data)**	Slope (*p*-value)	0.67(<0.0001)	Slope (*p*-value)	0.68(<0.0001)
	Intercept(*p*-value)	-1.99(0.039)	Intercept(*p*-value)	-3.51(1.05)
	R^2^	0.842	R^2^	0.851
**Ambient PM**_**2.5**_ **(24-h average data)**	Slope (*p*-value)	0.73(<0.0001)	Slope (*p*-value)	0.71(<0.0001)
	Intercept(*p*-value)	-5.62(<0.0001)	Intercept(*p*-value)	-5.86(<0.0001)
	R^2^	0.910	R^2^	0.901

The slopes in the indoor-ambient PM_2.5_ regression models can be the estimates of the infiltration factors of ambient PM_2.5_ (*F*_inf_)[25, 27], whereas the intercepts can be the estimates of the concentrations of indoor PM_2.5_ generated by indoor sources[25, 27]. The values of *F*_inf_, which represented the fraction of infiltrated ambient PM_2.5_ that remained suspended[25], were 0.67–0.73 on the community basis (Table 3); whereas the *F*_inf_ of single residences largely varied from 0.18 to 0.90. The modeling analyses yielded negative intercepts (p<0.05, Table 3). The negative estimates implied that particles of indoor origin only accounted for a small fraction of residential indoor PM_2.5_ compared to that infiltrated from outdoors. On the other hand, there may be significant sink of infiltrated ambient PM_2.5_ in the indoor spaces. According to a balance-point concept developed by Li and Chen[45], a balance-point outdoor PM_2.5_ concentration is present for a given indoor microenvironment [45]. When outdoor PM_2.5_ concentration equals the balance-point one, a balance state is reached between indoor particle generation speed and particle deposition rate, under which I/A of PM_2.5_ equals penetration efficiency [45]. When outdoor concentration exceeds the balance-point outdoor value, I/A is lower than penetration efficiency, implying net decay of infiltrated PM_2.5_. The ambient PM_2.5_ concentrations were generally higher than the balance-point value under the distinctive high ambient fine particle pollution in Beijing, and the net decay (sink) of infiltrated PM_2.5_ was expected. The net decay of infiltrated PM_2.5_ resulted in the lower I/A ratio in our tests, the mean and median of which were 0.63 and 0.62, respectively.

The indoor/ambient ratio (I/A) of PM_2.5_ mass concentration is another important indicator of indoor-outdoor relationship of PM_2.5_. A high I/A is usually associated with significant indoor PM_2.5_ sources. Thus, I/A can be used to examine the impacts of indoor sources on indoor PM_2.5_ mass concentrations. As shown in Table 1, the mean I/A over the 24-h sampling period was 0.63±0.15 with median of 0.62. Peaks of I/A were found at 12:00–13:00 and 23:00–0:00 (Fig 2), suggesting the presence of significant indoor PM_2.5_ sources in the two time intervals. In addition, slight increase of I/A can be discerned in early morning (7:00–9:00) and evening (15:00–17:00) (Fig 2).

### Predictive models of indoor and ambient PM_2.5_ mass concentrations

In this study, the OLS method was utilized to examine the associations between a variety of factors and PM_2.5_ mass concentrations.

The variables considered for the predictive model of ambient PM_2.5_ mass concentrations were ambient criteria air pollutant concentrations. The results, which are presented in Table 4, indicated that ambient PM_2.5_ concentrations were positively associated (p<0.05) with the concentrations of ambient O_3_, NO_2_ and SO_2_. The three pollutants contributed to 7.4% of total variance of ambient PM_2.5_ mass concentrations.

**Table 4:** Predictive model of ambient PM_2.5_ mass concentrations.

Variable		Ambient PM_2.5_
**O**_**3**_	Slope	0.075
	*p*-value	0.040
	R^2^	0.006
**NO**_**2**_	Slope	0.42
	*p*-value	<0.001
	R^2^	0.021
**SO**_**2**_	Slope	1.61
	*p*-value	<0.0001
	R^2^	0.047

The variables considered for the model of indoor PM_2.5_ mass concentrations were subject to built environment, human behavior and chemistry. As shown in Table 5, there were 10 variables entering the predictive model of all families. The predictive model explained 41.1% of total variance. The variable having the maximum variance contribution was *number of occupants* (21.5% of total variance and thereafter), followed by *ambient SO*_*2*_ (7.7%), *ambient O*_*3*_ (2.8%) and *ambient NO*_*2*_ (1.9%). The variables subject to lifestyle and built environment had relatively small variance contribution (Table 5).

**Table 5:** Predictive model of indoor PM_2.5_ mass concentrations.

	Indoor PM_2.5_ (including all families)			Indoor PM_2.5_ (excluding smoking families)		
Variable	Slope	*p*-value	R^2^	Slope	*p*-value	R^2^
Distance to road ^a^	-4.52	<0.0001	0.011	-2.11	0.095	0.003
Frequency of furniture cleaning ^b^	-12.9	<0.0001	0.016	N/A	N/A	N/A
Smoking ^c^	4.15	<0.01	0.008	N/A	N/A	N/A
Indoor plants ^d^	6.26	<0.0001	0.013	4.21	0.013	0.007
Frequency of floor cleaning ^e^	7.15	<0.0001	0.011	4.34	0.011	0.007
Floor cleaning method ^f^	7.15	<0.0001	0.013	6.14	0.029	0.005
Number of occupants	8.33	<0.0001	0.215	10.26	<0.0001	0.286
Traffic route ^g^	N/A	N/A	N/A	-4.38	<0.0001	0.030
Cooking	N/A	N/A	N/A	-1.78	0.135	0.002
O_3_	0.37	<0.0001	0.028	0.077	0.045	0.004
NO_2_	0.82	<0.0001	0.019	0.29	0.091	0.003
SO_2_	2.04	<0.0001	0.077	1.86	<0.0001	0.079

a. the levels of *distance to road*: 1- ≤ 193 m, 2–194–270 m, 3–271–425 m, 4- >425 m.

b. the levels of *frequency of furniture cleaning*: 1- everyday, 2- some times per week, 3- one time for several weeks.

c. the levels of *smoking*: 0- no smoking, 1- yes, 1time, 2- yes, multiple times.

d. the levels of *indoor plants*: 1- no plants, 2- a few, 3- many.

e. the levels of *frequency of floor cleaning*: 1- everyday, 2- some times per week.

f. the levels of *floor cleaning method*: 1- only mop, 2- with mop and broom/vacuum.

g. the levels of *traffic route*: 1-arterial road (ring roads and express ways), 2-urban highway, 3-quiet street.

The predictive model of the non-smoking families was presented in Table 5 as well. The model explained 42.4% of total variance. Compared to the model of all families, the variables, *traffic route* and *cooking*, were included in the model, while the variable, *frequency of furniture cleaning*, was not included. The variable having the maximum variance contribution was still *number of occupants* (28.6%), followed by *ambient SO*_*2*_ (7.9%), *traffic route* (3.0%), *indoor plants* (0.7%), *frequency of floor cleaning* (0.7%), *floor cleaning method* (0.5%), *ambient O*_*3*_ (0.4%), *ambient NO*_*2*_ (0.3%), *distance to the road* (0.3%) and *cooking* (0.2%).

## Discussion

The mean ambient PM_2.5_ concentration over 24-h period in Beijing was 69 μg/m^3^ in this study; while the counterpart in the North American metropolitan areas such as Los Angeles, Boston and Toronto were as low as 25, 14 and 28 μg/m^3^, respectively [30, 46, 47]. Additionally, the outdoor PM_2.5_ mass concentrations over 24-h or 48-h period in several large-scale studies such as the RIOPA and EXPOLIS were reported to be 14–21 and 10–40 μg/m^3^, respectively [21, 33]. It can be seen that the ambient PM_2.5_ mass concentrations in Beijing were much higher than those in the US and Europe. The sampling was conducted from July to September, during which the windows were open most of time. Given such, ambient PM_2.5_ may strongly influence residential indoor PM_2.5_ under the high ambient fine particle pollution conditions. This statement is supported by the Pearson correlation analysis results, which showed significantly strong (r≥0.90) and positive correlation between ambient and indoor PM_2.5_ data. The strong impact of ambient PM_2.5_ on the indoor counterpart is also demonstrated by the regression analysis results, which showed that ambient PM_2.5_ mass concentration data contributed >80% total variance of indoor PM_2.5_ data. The results also implied that PM_2.5_ of indoor origin only accounted for a small fraction of indoor PM_2.5_ in this study. According to these results, PM_2.5_ of ambient origin made dominant contribution to residential indoor PM_2.5_ exposure in the non-heating season. The synchronous diurnal variation of ambient and indoor PM_2.5_ mass concentrations was thus within expectation as well. The dominant contribution of ambient PM_2.5_ does not necessarily mean that it was reasonable to use ambient PM_2.5_ mass concentration as the surrogate of residential indoor PM_2.5_ exposure in the non-heating season. The inter-residence variability of *F*_inf_ values indicated that the infiltration efficiency of ambient PM_2.5_ largely varied by dwellings. As a result, the use of ambient PM_2.5_ mass concentrations as the exposure surrogates can lead to significant exposure misclassification.

The diurnal variation pattern of ambient PM_2.5_ mass concentrations in our study was very similar to that observed in Windsor, Canada (referred as the Windsor study and thereafter)[39]. The similarity implies sharing mechanisms underlying ambient PM_2.5_ mass concentration variation in the two studies. The morning increase of ambient PM_2.5_ concentrations (5:00–8:00) was attributed to heavy traffic emission in the morning “rush” hours (Fig 2). The morning increase was also observed in the Windsor study [39] and the retirement homes in Los Angeles Basin (referred as the Los Angeles study and thereafter)[30]. The falling ambient PM_2.5_ concentrations in 10:00–17:00 were accompanied with substantially increasing ambient O_3_ concentrations (Fig 2). The substantially increasing O_3_ concentrations in ambient air resulted from the intense photochemical reactions in the summer. The intense photochemical reactions promote formation of secondary particulate matter in ambient PM[30], which are usually in ultrafine size bins[20, 39]. Several studies have observed substantially increased concentrations of ambient ultrafine PM (UFP) in the afternoon [20, 39]. However, UFP only have small contribution to PM_2.5_ mass concentrations. As a result, the increased ambient O_3_ concentrations in the afternoon did not lead to increasing ambient PM_2.5_ mass concentrations in our tests as well as the Windsor and Los Angeles studies[30, 39]. The evening increase of ambient PM_2.5_ concentrations was possibly driven by two mechanisms: a) heavy traffic emission in the evening “rush” hours; b) a lower mixing height in atmosphere, which does not favor diffusion and dispersion of air pollutants [30]. The lower mixing height in atmosphere resulted in the higher concentrations at 21:00–3:00, and the elevating mixing height since 3:00 was the likely cause of the slight concentration decrease in 3:00–5:00. The higher mixing height resulted in the lower concentrations in the late morning and afternoon.

There was noticeable increase of I/A at 23:00–0:00, indicating the presence of significant indoor PM_2.5_ sources at this time interval. The increase was likely due to intensive indoor human activities in the evening, when there were maximum occupants indoors. We acknowledge that these activities could not be identified from the questionnaires. Intensive indoor human activities, such as cooking, were one of the probable factors leading to the noticeable increase of I/A at 11:00–13:00. Indoor human activities, such as cooking and indoor cleaning, may contribute to the slight increase of I/A at 7:00–9:00 and 15:00–17:00. Another likely contributor of the slight increase at 7:00–9:00 was fresh traffic emission. The fresh traffic emission in the morning rush hours resulted in higher elemental carbon (EC) concentrations in atmosphere and smaller size distribution[22, 30]. The outdoor-to-indoor infiltration during this period was expected to higher, and thus a slight increase of I/A was within expectation.

The predictive models in this study can help characterizing possible sources of airborne particles. It is known that gas-phase oxidation of ambient SO_2_ and NO_2_ induced by atmospheric hydroxyl radical produces sulfate and nitrate particles, which are in accumulation mode [31, 44]. Ambient O_3_ are associated with the formation of secondary organic carbons in accumulation mode, because both are products of photochemical reactions in atmosphere [44]; whereas O_3_ can also contribute to formation of secondary organic carbons [35, 48]. It is thus reasonable that ambient PM_2.5_ mass concentrations were positively associated with the concentrations of ambient SO_2_, NO_2_ and O_3_. The associations suggested the contribution of secondary inorganic and organic aerosols to ambient PM_2.5_.

The concentrations of ambient SO_2_, NO_2_ and O_3_ were also positively associated with the indoor PM_2.5_ concentrations (Table 5). The associations implied the significant contribution of secondary aerosols to indoor PM_2.5_. The secondary aerosols included those infiltrated from outdoors, and also included those formed indoors via O_3_-terpene reactions [35, 48]. The associations between *distance to traffic road* and indoor PM_2.5_ mass concentrations (Table 5) suggested that aerosols related to traffic emission contributed to indoor PM_2.5_ and the influence was distance dependent. It is necessary to note that the actual association between indoor PM_2.5_ mass concentration and *distance to the road* was not linear relationship. In the companion article studying the determinants on infiltration efficiency of ambient PM_2.5_, we found that the infiltration efficiency reached highest within the 2^nd^ quartile distance and lowest within the 4^th^ quartile distance during our field tests. The variable, *proximate traffic route type*, entered the model of non-smoking families (Table 5). A higher indoor PM_2.5_ concentration was associated with the buildings proximate to arterial road (ring roads), and a lower concentration was associated with the buildings proximate to quiet street. The results are reasonable given that the traffic emission was stronger in the arterial roads than in the quiet streets.

The variance of indoor PM_2.5_ data that remained unexplained by ambient PM_2.5_ indicated the potential implication of indoor PM_2.5_ sources. The predictive models of indoor PM_2.5_ mass concentrations characterized some of these sources. **Indoor plants** could contribute to indoor PM_2.5_ through resuspension of soil particles and formation of secondary submicron particles via ozone-biogenic terpenes reactions [35, 48]. Thus, more plants in residential indoor spaces was found to result in higher indoor PM_2.5_ mass concentrations in our study (Table 5).

As earlier discussed, ambient PM_2.5_ mass concentrations were generally higher than the balance-point values under the distinctive ambient fine particle pollution conditions. As a result, significant sink (net decay) of infiltrated ambient PM_2.5_ was expected in our study. The fine particles can deposit on various indoor surfaces. **Indoor cleaning activities** can lead to resuspension of particles [37, 38]. Therefore, the variables such as *frequency of furniture cleaning*, *frequency of floor cleaning* and *floor cleaning method* in this study were found to be the predictors of indoor PM_2.5_ concentrations. Note that the OLS analysis showed different relationship patterns for frequency of floor cleaning and frequency of furniture cleaning. Less frequently the occupants cleaned the floor, higher the indoor PM_2.5_ concentrations were, and it was reverse for the influence of furniture cleaning frequency. The mechanisms driving the differences are not clear. Moreover, indoor PM_2.5_ mass concentrations were higher in the residences where occupants cleaned floor with mop and broom/vacuum than in those where occupants cleaned floor with mop only. It is within expectation because cleaning with broom or vacuum can lead to more resuspension of particles.

**Smoking** was identified as another indoor PM source in this study. As shown in the predictive model of all families (Table 5), the indoor PM_2.5_ concentrations were higher in the residences where smoking activities occurred for at least one time. It is interesting that **cooking** was only found to be an identified indoor PM_2.5_ source in the non-smoking families (Table 5). In addition, cooking activities did not lead to sharp increase of I/A in our field tests. The results suggested that the associations between cooking and indoor PM_2.5_ mass concentrations were not as strong as those of other factors. One possible reason is that occupants turned on the hood and possibly isolated kitchen microenvironment with other spaces in residence via closing doors when cooking. Another possible reason is that particles generated by cooking are mainly in ultrafine size bins[39], which only account for small fraction of PM_2.5_ mass concentration.

The variable, *number of occupants*, had greater contribution to total variance of indoor PM_2.5_ data than other variables. A higher number of occupants indoors are usually associated with more intensive human activities indoors. More intensive human activities could generate more indoor fine particles. Nonetheless, the specific human activities indoors that generated PM_2.5_ were not clear. These activities may include, but not limited to, the activities related to the other variables in the predictive model.

## Conclusions and Recommendation

Ambient and residential indoor PM_2.5_ mass concentrations were concurrently collected for 41 residences in urban area of Beijing in the non-heating season. Ambient PM_2.5_ mass concentrations in Beijing were much higher than those in other metropolitan areas in the US and Europe. The high ambient fine particle pollution condition resulted in the following observations in this study: a) the median indoor/ambient ratio of PM_2.5_ mass concentration was as low as 0.63; b) ambient and residential indoor PM_2.5_ concentrations highly correlated with each other; c) ambient PM_2.5_ mass concentrations explained >80% total variance of indoor data. According to the results, ambient PM_2.5_ dominated residential indoor exposure to PM_2.5_ in the non-heating season on the community basis. However, it is still not reasonable to use ambient PM_2.5_ mass concentrations as the exposure surrogates in non-heating season, given the large inter-residence variability of infiltration efficiency of ambient PM_2.5_. On the other hand, PM_2.5_ of indoor origin still had minor influence on indoor PM_2.5_ mass concentrations. Noticeable indoor PM_2.5_ sources were identified at 11:00–13:00 and 22:00–0:00. The predictive models suggested that particles from traffic emission, secondary aerosols, particles from indoor smoking, suspended particles due to indoor cleaning and particles related to indoor plants contributed to indoor PM_2.5_ mass concentrations in this study.

The hourly real-time PM_2.5_ mass concentration data were collected in this study. The data were subsequently used to characterize the diurnal variation of ambient and indoor PM_2.5_ mass concentrations, in an effort to identify the time intervals in which there were significant indoor PM_2.5_ sources. On the other hand, emissions of many indoor sources, such as smoking, cooking and cleaning, are instantaneous or last for a short period. Emissions of these sources can influence indoor PM_2.5_ concentrations via generation of concentration spikes. However, the impacts of concentration spikes cannot be fully reflected in the diurnal variation pattern characterized by the hourly data. It is thus recommended to use data of higher time resolution in the future, which can favor characterizing the influence of indoor sources on indoor PM_2.5_ mass concentrations.

There is a delay period before equilibrium is reached between indoor PM_2.5_ and the infiltrated fraction. The delay period can be influenced by a variety of factors, an important of which is ventilation condition. The ventilation condition is an important determinant on the indoor-ambient relationship of PM_2.5_ mass concentrations. However, the real-time hourly ventilation rates of the tested residences were not measured in this study. As a result, we did not characterize the influence of ventilation condition on the indoor-ambient relationship. This is another major limitation of this study.

Real-time ventilation condition is a sophisticated function of many variables subject to building materials, building envelope structure, interior layout and human activities, so does the indoor-ambient relationship of PM_2.5_ mass concentrations. Nevertheless, only limited variables were considered in this study. The very incompleteness led to ~60% variance of indoor PM_2.5_ data that remained unexplained by the predictive models. On the other hand, we found 8 residences in this study, where indoor and ambient PM_2.5_ mass concentrations had weak correlation with each other. The regression analysis results suggested that there were potential strong indoor PM_2.5_ sources in these residences. However, any human activities related to the potential indoor PM_2.5_ sources could not be identified through the questionnaire. Given such, improvement of the questionnaire is recommended for the prospective similar study.

It is found that the indoor PM_2.5_ mass concentrations were associated with the distance to road in this study. The association resulted from the alteration of PM properties during aging and transport, such as size distribution and PM speciation. The property alteration can influence the infiltration behavior of ambient PM_2.5_, and thus influence the I/A. Therefore, size distribution and PM speciation can be more straightforward predictors of I/A. However, we did not collect PM properties in this study. It is recommended that PM properties are measured in the future and are used to build up relevant predictive models for indoor and ambient PM_2.5_ mass concentrations. The results may greatly contribute to the knowledge of residential indoor exposure to ambient PM_2.5_.

Moreover, Beijing has four distinctive seasons, and meteorological conditions, urban ambient pollution and human activity pattern dramatically differ in the heating and non-heating seasons. Thus, it is recommended to study the indoor-outdoor relationship of PM_2.5_ mass concentrations in the heating season as part of fine particle exposure assessment.

## Supporting Information

S1 File Table A- Residence locations and nearest environmental quality monitoring stations (EQM); Table B- Comparison of hourly ambient PM_2.5_ mass concentrations provided by EQM and measured by the light-scattering devices in our study; Fig A- Tests of normal distribution hypotheses for ambient PM_2.5_ data; Fig B- Tests of normal distribution hypotheses for indoor PM_2.5_ data; Fig C- Tests of normal distribution hypotheses for ambient O_3_ data; Fig D- Tests of normal distribution hypotheses for ambient NO_2_ data; Fig E- Tests of normal distribution hypotheses for ambient SO_2_ data. (DOCX)
