# Supplementary material for: Characterizing the Indoor-Outdoor Relationship of Fine Particulate Matter in Non-Heating Season for Urban Residences in Beijing
Source: PLoS One. 2015 Sep 23;10(9):e0138559. doi: 10.1371/journal.pone.0138559 (PMC4580321; doi:10.1371/journal.pone.0138559)
Supplement: S1 File — (DOCX) [file pone.0138559.s001.docx]

S1 Supplemental Information

TABLE S1 in S1. Residence locations and nearest environmental quality monitoring stations(EQM)

| Home ID | Location | Nearest CHEPA station | Distance  (km) | Home ID | Location | Nearest CHEPA station | Distance  (km) |
| --- | --- | --- | --- | --- | --- | --- | --- |
| H01 | Haidian | Wanliu | 1.5 | H22 | Fengtai | Wanshouxigong | 5.0 |
| H02 | Haidian | Wanliu | 7.0 | H23 | Xicheng | Guanyuan | 0.8 |
| H03 | Haidian | Wanliu | 6.2 | H24 | Chaoyang | Tiantan | 4.1 |
| H04 | Haidian | Wanliu | 3.9 | H25 | Xicheng | Guanyuan | 1.0 |
| H05 | Haidian | Wanliu | 4.3 | H26 | Chaoyang | Olympic center | 2.2 |
| H06 | Haidian | Wanliu | 5.5 | H27 | Xicheng | Guanyuan | 2.3 |
| H07 | Haidian | Wanliu | 3.2 | H28 | Chaoyang | Olympic center | 6.0 |
| H08 | Haidian | Wanliu | 5.5 | H29 | Changping | Changping Town | 1.6 |
| H09 | Haidian | Wanliu | 3.7 | H30 | Chaoyang | Olympic center | 2.7 |
| H10 | Haidian | Guanyuan | 2.2 | H31 | Chaoyang | Olympic center | 5.9 |
| H11 | Haidian | Wanliu | 3.0 | H32 | Chaoyang | Olympic center | 5.9 |
| H12 | Haidian | Wanliu | 3.8 | H33 | Chaoyang | Olympic center | 1.5 |
| H13 | Haidian | Wanliu | 5.5 | H34 | Chaoyang | Agri. Exhibit. Ctr. | 4.6 |
| H14 | Haidian | Wanliu | 6.2 | H35 | Chaoyang | Olympic center | 6.4 |
| H15 | Fengtai | Gucheng | 5.2 | H36 | Shijingshan | Gucheng | 2.5 |
| H16 | Chaoyang | Olympic center | 6.0 | H37 | Haidian | Wanliu | 3.8 |
| H17 | Chaoyang | Agri. Exhibit. Ctr. | 6.0 | H38 | Haidian | Olympic center | 5.4 |
| H18 | Chaoyang | Agri. Exhibit. Ctr. | 5.7 | H39 | Haidian | Wanliu | 3.2 |
| H19 | Fengtai | Gucheng | 5.2 | H40 | Chaoyang | Olympic center | 5.2 |
| H20 | Xicheng | Guanyuan | 1.9 | H41 | Chaoyang | Agri. Exhibit. Ctr. | 4.8 |
| H21 | Xicheng | Guanyuan | 2.5 |  |  |  |  |

TABLE S2 in S1. Comparison of hourly ambient PM_2.5_ mass concentrations provided by EQM and measured by the light-scattering devices in our study

| Nearest EPA station name | Monitoring period | Distance (km) | 95% CI of differences | Pearson correlation coefficient |
| --- | --- | --- | --- | --- |
| Wan Liu | 8 hours | 4.7 | (-2, 3) μg/m^3^ | 0.91 |
| Wan Liu | 10 hours | 1.6 |  | 0.86 |
| Olympics Center | 5 hours | 5.2 |  | 0.92 |
| Agriculture Exhibition Center | 16 hours | 2.2 |  | 0.95 |
| Guan Yuan | 11 hours | 7.9 | (-9, 8) μg/m^3^ | 0.94 |
| All data | N/A | N/A | (-2, 2) μg/m^3^ | N/A |

Figures


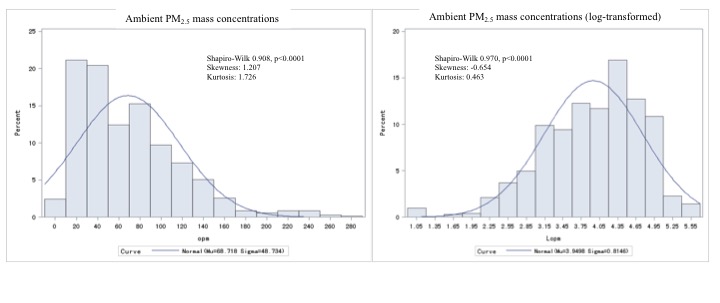


Figure S1 in S1. Tests of normal distribution hypotheses for ambient PM_2.5_ data


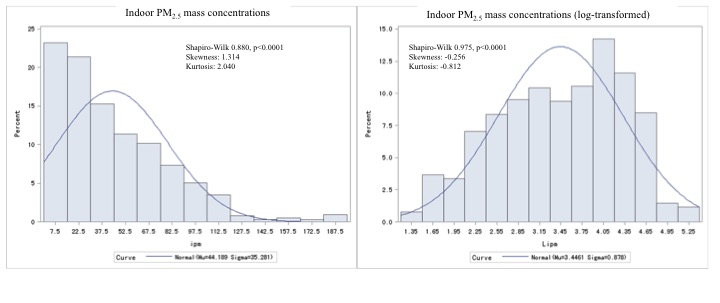


Figure S2 in S1. Tests of normal distribution hypotheses for indoor PM_2.5_ data


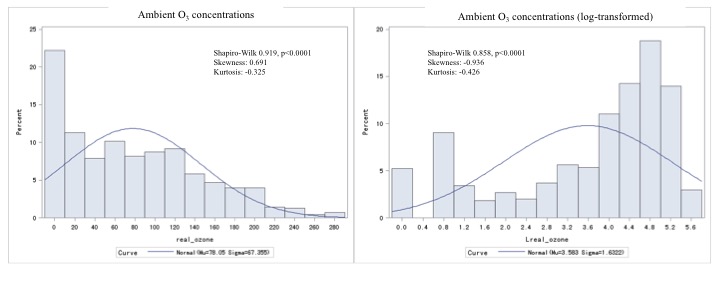


Figure S3 in S1. Tests of normal distribution hypotheses for ambient O_3_ data


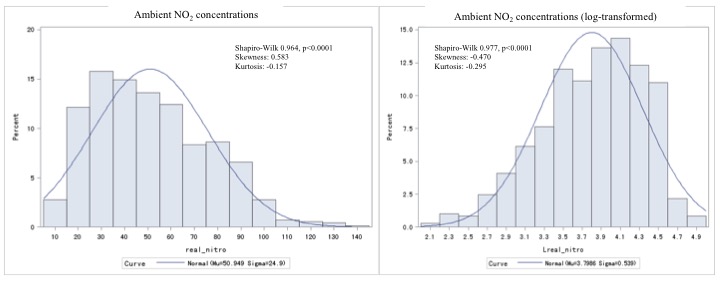


Figure S4 in S1. Tests of normal distribution hypotheses for ambient NO_2_ data


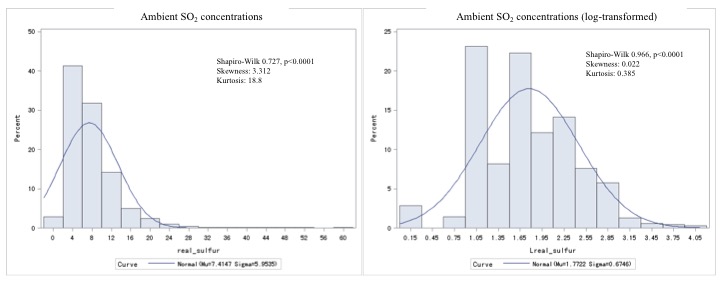


Figure S5 in S1. Tests of normal distribution hypotheses for ambient SO_2_ data
